# Luteolin alleviates inflammation and autophagy of hippocampus induced by cerebral ischemia/reperfusion by activating PPAR gamma in rats

**DOI:** 10.1186/s12906-022-03652-8

**Published:** 2022-07-01

**Authors:** Lu Li, Guanghua Pan, Rong Fan, Dalei Li, Lei Guo, Lili Ma, Hui Liang, Jiaoxue Qiu

**Affiliations:** 1 Department of Electrophysiology, Weihai Municipal Hospital, Cheeloo College of Medicine, Shandong University, Weihai, 264200 China; 2 Department of Gastroenterology, Weihai Municipal Hospital, Cheeloo College of Medicine, Shandong University, Weihai, 264200 China; 3 Yantai Raphael Biotechnology Co.,Ltd, Yantai, 264200 China; 4 School of Pharmacy, Key Laboratory of Molecular Pharmacology and Drug Evaluation (Yantai University), Ministry of Education, Collaborative Innovation Center of Advanced Drug Delivery System and Biotech Drugs in Universities of Shandong, Yantai University, Yantai, 264000 China; 5 Department of Neurology, Yantaishan Hospital, No.10087, Keji Avenue, Laishan District, Yantai, 264000 China

**Keywords:** Cerebral ischemia-reperfusion, Pioglitazone, T0070907, Molecular docking analysis

## Abstract

**Background:**

Luteolin, a flavonoid compound with anti-inflammatory activity, has been reported to alleviate cerebral ischemia/reperfusion (I/R) injury. However, its potential mechanism remains unclear.

**Methods:**

The binding activity of luteolin to peroxisome proliferator-activated receptor gamma (PPARγ) was calculated via molecular docking analysis. Rats were subjected to middle cerebral artery occlusion and reperfusion (MCAO/R). After reperfusion, vehicle, 25 mg/kg/d luteolin, 50 mg/kg/d luteolin, 10 mg/kg/d pioglitazone, 50 mg/kg/d luteolin combined with 10 mg/kg/d T0070907 (PPARγ inhibitor) were immediately orally treatment for 7 days. ELISA, TTC staining, H&E staining, immunohistochemistry, immunofluorescence and transmission electron microscope methods were performed to evaluate the inflammation and autophagy in damaged hippocampal region. The PPARγ, light chain 3 (LC3) B-II/LC3B-I and p-nuclear factor-κB (NF-κB) p65 proteins expression levels in damaged hippocampal region were analyzed.

**Results:**

Luteolin showed good PPARγ activity according to docking score (score = − 8.2). Luteolin treatment downregulated the infarct area and the pro-inflammatory cytokines levels caused by MCAO/R injury. Moreover, luteolin administration ameliorated neuroinflammation and autophagy in damaged hippocampal region. Pioglitazone plays protective roles similar to luteolin. T0070907 concealed the neuroprotective roles of 50 mg/kg/d luteolin.

**Conclusions:**

Luteolin exerts neuroprotective roles against inflammation and autophagy of hippocampus induced by cerebral I/R by activating PPARγ in rats.

**Supplementary Information:**

The online version contains supplementary material available at 10.1186/s12906-022-03652-8.

## Background

Ischemic stroke is a critical and debilitating disease that leads to high death rate and long-term disability. It accounts for 85% of all stroke [1, 2], and is caused by blocking the flow of blood to the brain [2]. Restoring blood flow is essential for the treatment of ischemic stroke, but reperfusion itself may lead to additional injury, called ischemia reperfusion (I/R) injury [3]. Anomalous permeability of the blood brain barrier and risk of hemorrhagic conversion were increased by reperfusion. Current treatments for ischemic stroke are mainly mechanical thrombectomy and/or the treatment of athrombolytic drugs [2, 4]. Therefore, more effective drugs for ischemic stroke are need to keep trying and exploring.

Neuroinflammation acts a key event in the immune defense by increasing proinflammatory mediators, activating microglia, and increasing the proliferation of various types of inflammatory cells after cerebral I/R [5]. It is known that traumatic brain injury results in the dysregulation of autophagy and contributes to neuronal death in damaged brain [6, 7]. In cerebral I/R injury, researchers found that autophagy protected against inhibition of the inflammatory response, and the extent of protection was related with the stage of the cerebral I/R injury and the level of autophagy response [8]. Activation of peroxisome proliferator-activated receptors (PPARs) has shown neuroprotective effects in different neurodegenerative diseases including cerebral I/R [9–11]. Activation of PPAR subtypes, especially PPAR gamma (PPARγ), has been shown to prevent neuronal damage and inflammation in cerebral I/R injury through inhibiting the secretion of inflammatory cytokines, such as interleukins (IL)-1β and tumor necrosis factor (TNF)-α, and stimulating nuclear factor-κB (NF-κB) activation thereby attenuating neuronal autophagic death [12, 13]. Thus, activation of PPARγ is a potential therapeutic approach for cerebral I/R damage.

Luteolin (3′,4′,5,7-tetrahydroxy flavone), a dietary flavone, is found in different plants and has a C6-C3-C6 structure containing two benzene rings and one oxygen-containing ring with a C2-C3 carbon double bond [14, 15]. It has been reported that luteolin could diminish infarct size and neutrophil accumulation in the ischemic myocardium [12, 16, 17]. In addition, luteolin has anti-inflammatory and neuroprotective effects in age-related neurodegenerative disorders, for example Alzheimer’s disease, Parkinson’s disease, diabetes-associated cognitive decline and traumatic brain injury [15]. Luteolin exerts good regulatory effects on antioxidant and neuroinflammation through inhibiting reactive oxygen species (ROS) and inflammatory cytokines in cerebral I/R damage. In particular, Li and colleagues [12] found that luteolin could regulate PPARγ to attenuate neuroinflammation in focal cerebral ischemia. Taken together, it is reasonable to presume luteolin is closely involved in the anti-inflammatory response through PPARγ after cerebral I/R damage.

In this study, we predicted that PPARγ is a target gene of luteolin through the Traditional Chinese Medicine Systems of Pharmacology Database and Analysis Platform (TCMSP, https://tcmsp-e.com), and luteolin showed good PPARγ activity (score = − 8.2) through UCSF chimera software (https://www.cgl.ucsf.edu/chimera/). It provides a good theoretical basis for analyzing the neuroprotective mechanism of luteolin in rats after middle cerebral artery occlusion and reperfusion (MCAO/R) injury.

## Methods

### Molecular docking analysis

The 3D structure of the luteolin ligand was retrieved from PubChem (https://pubchem.ncbi.nlm.nih.gov/), and the three-dimensional structure of PPARγ was downloaded from the PDB database (https://www.rcsb.org/). According to a reported study [18], mechanical optimization, hydrogenation and charging of the ligand were carried out by UCSF chimera software (https://www.cgl.ucsf.edu/chimera/). The AutoDock Vina tool was performed to obtain molecular docking. A grid box was produced that was large enough to cover the entire protein binding site and allow all ligands to move freely. Total-score represents the docking result.

### Animals

Seventy-two Sprague Dawley male rats, 200–220 g, 5–6 weeks old, were obtained (Jinan Pengyue Experimental Animal Breeding Co., Ltd., China) and housed under a 12 h light/dark cycle at 20–24 °C with 40–70% relative humidity. The rats were adapted for 1 week with access to food and water ad libitum*.*

### MCAO/R model

As previous report [1], rats were intraperitoneally anesthetized with 1.5% pentobarbital sodium (0.27 mL / 100 g). After exposing the bilateral common carotid arteries, the right common carotid artery (CCA), external carotid artery (ECA), and internal carotid artery (ICA) were discriminated. An intraluminal filament was inserted into the ICA (17–19 mm) through the ECA stump for 120 min. Then, the filament was put out to start reperfusion, carefully.

The sham operated rats underwent the same procedure without filament occlusion.

### Experimental groups

The rats were divided into six groups (12 in each group) as follows: 1) sham operated group (sham); 2) MCAO/R (vehicle) group, rats underwent MCAO/R and were then orally treated with 0.9% sterile saline for 7 days at beginning of reperfusion; 3) luteolin-25 group, rats underwent MCAO/R and were then orally treated with 25 mg/kg/d luteolin (MFCD00017309, Macklin, Shanghai, China) dissolved in 0.9% sterile saline (w/v) for 7 days at beginning of reperfusion [19, 20]; 4) luteolin-50 group, rats underwent MCAO/R and were then orally treated with 50 mg/kg/d luteolin dissolved in 0.9% sterile saline (w/v) for 7 days at beginning of reperfusion [19, 20]; 5) pioglitazone group, rats underwent MCAO/R and were then orally treated with 10 mg/kg/d PPARγ agonist pioglitazone (HY-13956, MedChemExpress, Shanghai, China) [21] dissolved in 0.9% saline for 7 days at beginning of reperfusion; 6) luteolin-50 + T0070907 group, rats underwent MCAO/R and were then orally treated with 50 mg/kg/d luteolin combined with 10 mg/kg/d PPARγ inhibitor T0070907 (HY-13202, MedChemExpress, Shanghai, China) [21] dissolved in 0.9% saline for 7 days at beginning of reperfusion.

### Enzyme-linked immunosorbent assays (ELISAs)

After anesthetization with 1.5% pentobarbital sodium, blood was taken from the abdominal aorta of the rats. The levels of IL-1β (bs-10859R, Bioss, China), IL-6 (bs-0781R, Bioss, China) and TNF-α (Bsk13003, Bioss, China) in the blood was detected by an ELISA kit at an absorbance of 450 nm.

### 2,3,5-Triphenyltet-razolium chloride (TTC) staining

All rats were anaesthetized and perfused with phosphate buffered saline (PBS) followed by 4% paraformaldehyde. After washing with 0.9% saline, the brain was frozen at − 20 °C for 10 min. Then, the brain tissue was cut into 6 sections, and soaked in 1% TTC solution (20,190,917, Solarbio, China) at room temperature for 15 min without light. The coronal slices were photographed and analyzed by Image J software (National Institutes of health, USA). Infarct area percent (%) = infarct area/total area × 100%.

### Hematoxylin-eosin (HE) staining

After fixing with 4% paraformaldehyde for 24 h, brain tissues were embedded in paraffin, and cut into slices (3 μm). After dewaxing with xylene, the sections were hydrated with different concentrations ethanol (100%, 5 min; 95%, 2 min; 80%, 2 min; 70%, 2 min). After that, the sections were stained with HE staining solution (G1120, Solarbio, China) for 30 min at 55 °C. After washing, the slices were separated with 95% ethanol for 1 min, and dehydrated with 100% ethanol for 2 min. After clearing with the xylene, the slices were sealed with neutral gum to observe under an optical microscope (DM1000 LED, Leica, Germany).

### Immunohistochemistry

The brain slices were heated to boiling in 0.01 mol/L sodium citrate buffer (pH = 6.0) using a microwave oven (2 times, an interval of 10 min 10 min), cooled at room temperature, and washed with 0.01 mol/L PBS (pH = 6.0) 3 times (5 min/wash). Then, the slices were cultured with 3% H_2_O_2_ for 10 min. The primary antibodies, including GFAP (#ab7260, 1:1000, Abcam, China) and Iba-1 (#ab178847, 1:100, Abcam, China) were cultured with the slices overnight at 4 °C. After washing with PBS, the goat secondary antibody (1:500, Thermo Fisher Science, China) was added to the culture for 60 min at 37 °C. After that, the slices were stained with diaminobenzidine (DAB) at 37 °C for 30 s and dehydrated, purified and sealed. The expressions of index were observed and photographed using an optical microscope. Positive protein expression was analysed using Image J software. Positive expression (%) = Positive area/total area × 100.

### Immunofluorescence

The brain slices (3 μm) were dewaxed and hydrated as descripted in HE staining. Then, the slices were heated to boiling in 0.01 mol/L sodium citrate buffer (pH = 6.0) using a microwave oven (2 times, an interval of 10 min), cooled at room temperature and washed with 0.01 mol/L PBS (pH = 6.0) for 3 times (5 min/wash). Then, the slices were cultured with 3% H_2_O_2_ for 10 min. The primary antibodies, including GFAP (#ab7260, 1:1000, Abcam, China), Iba-1 (#ab178847, 1:100, Abcam, China), LC3B (#ab63817, 1:1000, Abcam, China), NeuN (#ab104224, 1:1000, Abcam, China), were cultured with the slices for overnight at 4 °C. After washing, the goat secondary antibody (1:500, ThermoFisher Science, China) were added to culture for 60 min at 37 °C. After that, the slices were counterstained with 4′,6-diamidino-2-phenylindole (DAPI) at 37 °C for 5 min. After washing with PBS and quenching fluorescence, a laser confocal microscope (LSM800, Zeiss, Germany) was used to observe the results.

### Transmission electron microscope (TEM)

The damaged hippocampal region was fixed with electron microscope fixing solution (G1102, Servicebio) at 4 °C for 4 h. After washing with 0.1 M phosphate buffer (pH 7.4) 3 times, 15 min each time, 1% osmic acid 0.1 M phosphate buffer was added at 20 °C for 2 h. After washing, the tissues were dehydrated with an ethanol gradient (50–70%-80–90%-95–100%) followed by 100% ethanol for 15 min each time. Then, the tissues were embedded with acetone: 812 embedding agent (90529–77-4, SPI) =1: 1 for 4 h, and embedded with acetone: 812 embedding agent = 1: 2 for overnight. The samples were inserted into the embedding plate with 812 embedding agent at 37 °C overnight and 60 °C for 48 h. Slices (60 nm) were cut using ultra-thin slicer (Leica UC7, leica), and stained with 2% uranium acetate saturated alcohol solution for 15 min and lead citrate for 15 min. The slices were dried at room temperature and observed under TEM (HT7700, HITACHI).

### Western blot

The damaged hippocampal region tissues were separated with RIPA buffer (Beyotime, China) on ice and homogenized to extract protein. Forty μg proteins were separated with 12% SDS-PAGE (Bio-Rad, China) and transferred to PVDF membranes (EMD Millipore). After blocking with 5% milk for 1.5 h, the membranes were incubated with appropriate primary antibodies diluted with 5% BSA for overnight at 4 °C. The primary antibodies consisted of phospho-PPARγ (ser273) (1:1000, bs-4888R, Bioss, China), LC3BI/II (1:1000, 4108, Cell signaling technology, China), phospho-NF-κB p65 (1:800, bs-230303R, Bioss, China), GAPDH (1:1000, bs-0755R, Bioss, China). After washing with TBS-0.01% Tween 20 for 3 times (10 min/wash), the secondary antibody Goat Anti-rabbit lgG/HRP (1:1000, bs-0295G-HRP, Bioss, China) was cultured with the membranes for 2 h at 25 °C. After washing, the signals were visualized using enhanced chemiluminescence reagent (D085075, Bio-Rad, China).

### Statistical analysis

Data analysis was carried out with SPSS 20.0 (National Institutes of Health) software. The analysis results were expressed as mean ± standard deviation, and the differences among groups were analyzed using one-way analysis of variance (ANOVA), followed by Tukey post-test. *P* < 0.05 was considered significant.

## Results

### Luteolin binds to PPARγ and reduces the infarct area in MCAO/R treated rats

**Fig. 1:**
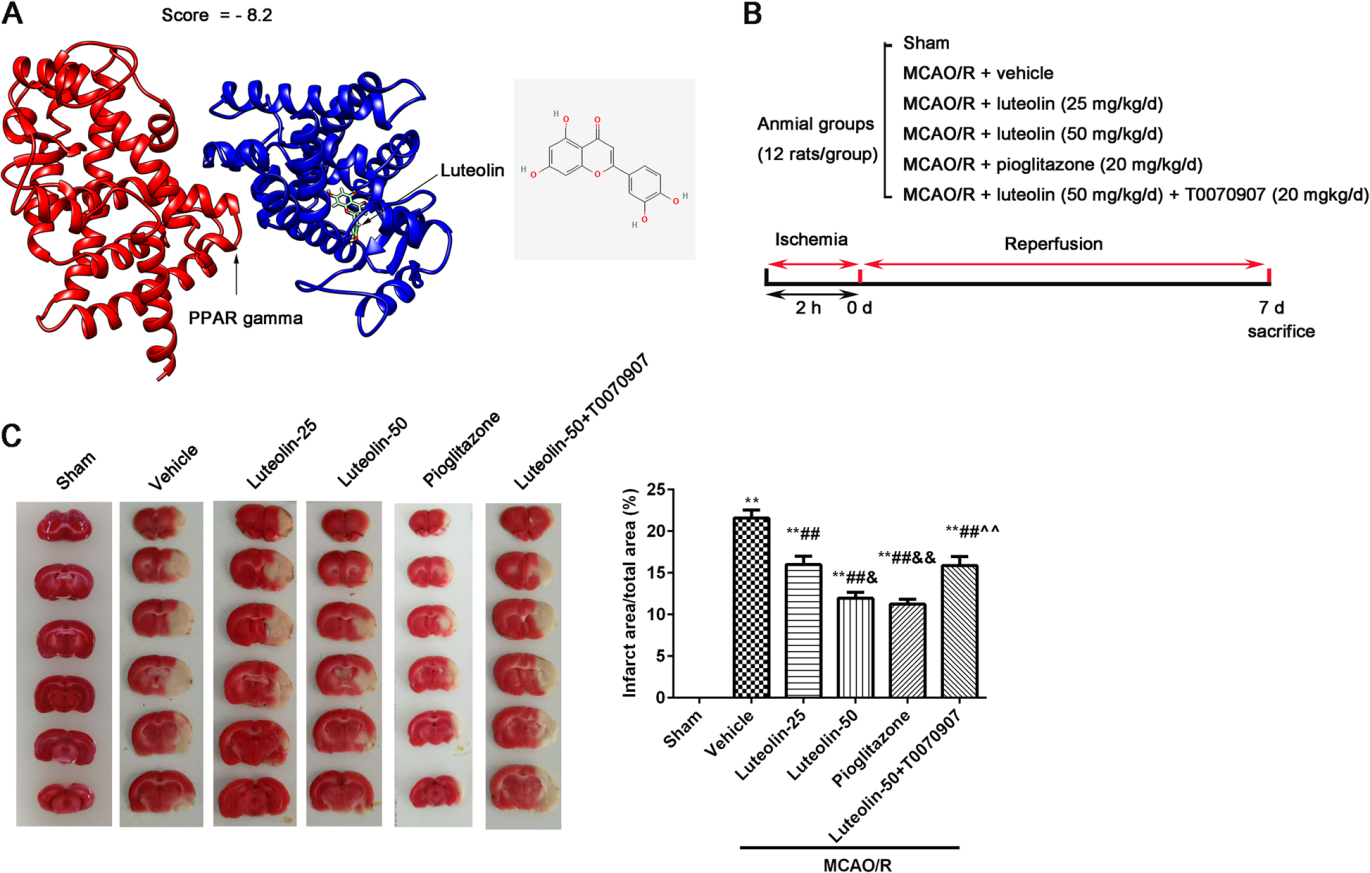
Luteolin binds to PPARγ and reduced the infarct area in MCAO/R treated rats. **A** The result of molecular docking; **B** The diagram of this experiment; **C** The infarct area was measured by TTC staining, and the infarct area (%) was counted through Image J software. Vs. sham group, ^**^p < 0.01; Vs. vehicle group, ^##^*p* < 0.01; Vs. luteolin-25 group, ^&^*p* < 0.05, ^&&^*p* < 0.01; Vs. luteolin-50 group, ^^^^*p* < 0.01

Total score indicates the inter molecular energy (kcal/mol), representing the stability between the ligand and receptor. The more negative the value is, the more stable the binding. As screening condition (the absolute value of a total score greater than 6.8), there was a good activity of luteolin against PPARγ (total score = − 8.2, Fig. 1A). In order to confirm luteolin improves brain injury induced by MCAO/R through PPARγ in rats, we designed this experiment (Fig. 1B). The brain infarct area was observed using TTC staining (Fig. 1C) and the infarct area is shown in white. From Fig. 1C, we found that 25 mg/kg/d luteolin and 50 mg/kg/d luteolin greatly reduced the infarct area when contrasted to the MCAO/R (vehicle) group. Interestingly, the results of pioglitazone were similar to the 50 mg/kg/d luteolin, but addition of T0070907 hampered the protective effect of 50 mg/kg/d luteolin.

### Luteolin improved hippocampal injury and reduced inflammatory factors through PPARγ in MACO/R treated rats

**Fig. 2:**
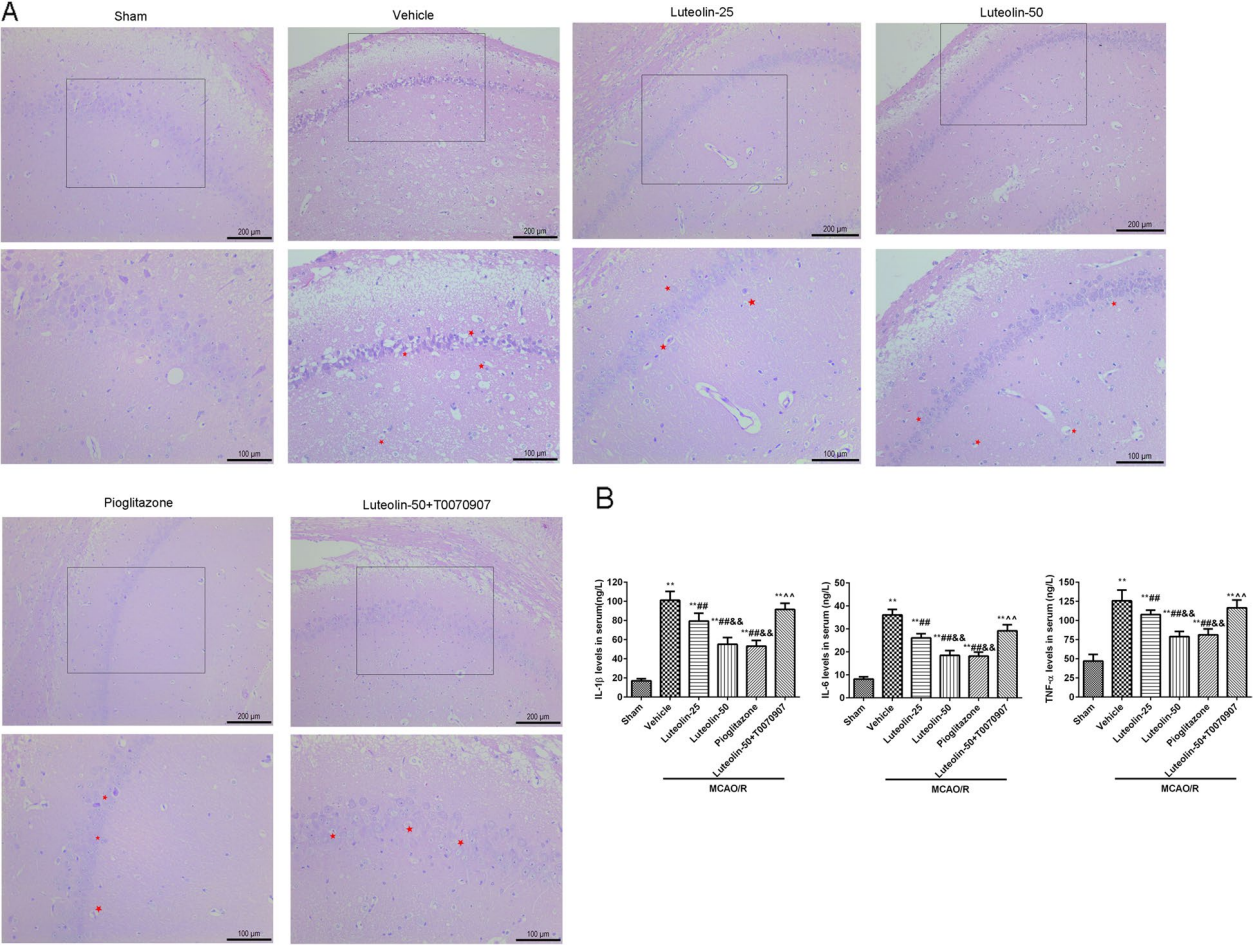
Luteolin improved hippocampal injury and reduced inflammatory factors through PPARγ in MCAO/R treated rats. **A** The changes of pathology in damagedCA1 hippocampal region were observed by HE staining, nerve cells shrink (red asterisks). **B** The levels of IL-1β, IL-6 and TNF-α in serum by ELISA. Vs. sham group, ^**^p < 0.01; Vs. vehicle group, ^##^*p* < 0.01; Vs. luteolin-25 group, ^&&^*p* < 0.01; Vs. luteolin-50 group, ^^^^*p* < 0.01

Changes of pathology in the damaged CA1 hippocampal region were observed through HE staining (Fig. 2A). MCAO/R resulted in a large of nerve cells shrink (red asterisks). After treatment with luteolin or pioglitazone, the numbers of nerve cells shrink decreased. Meanwhile, the protective effect of 50 mg/kg/d luteolin was suppressed by T0070907 administration. The inflammatory factor levels of IL-1β, IL-6 and TNF-α in serum (Fig. 2B) were obviously increased in other groups compared to the sham group. After administration of different doses of luteolin, the levels of the above pro-inflammatory factors were significantly decreased, and there was a significant difference between the luteolin-25 group and the luteolin-50 group. Compared with the luteilin-50 group, no difference was found in the pioglitazone group, but a significant difference was found in the luteilin-50 + T0070907 group.

### Luteolin reduced hippocampal neuroinflammation through PPARγ in MACO/R treated rats

**Fig. 3:**
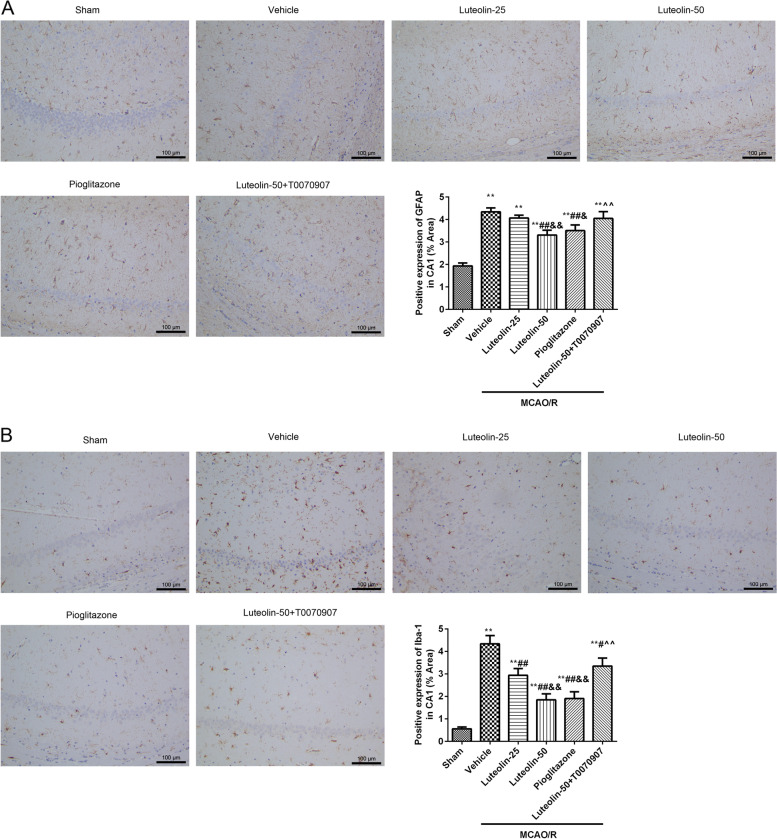
Luteolin suppressed GFAP and Iba-1 activation through PPARγ in damaged hippocampus in MCAO/R treated rats. The GFAP (**A**) and Iba-1 (**B**) expression levels in damaged CA1 hippocampal region were observed by immunohistochemistry. The positive expression was analyzed by Image J software. Vs. sham group, ^**^p < 0.01; Vs. vehicle group, ^#^*p* < 0.05, ^##^*p* < 0.01; Vs. luteolin-25 group, ^&^*p* < 0.05, ^&&^*p* < 0.01; Vs. luteolin-50 group, ^^^^*p* < 0.01

**Fig. 4:**
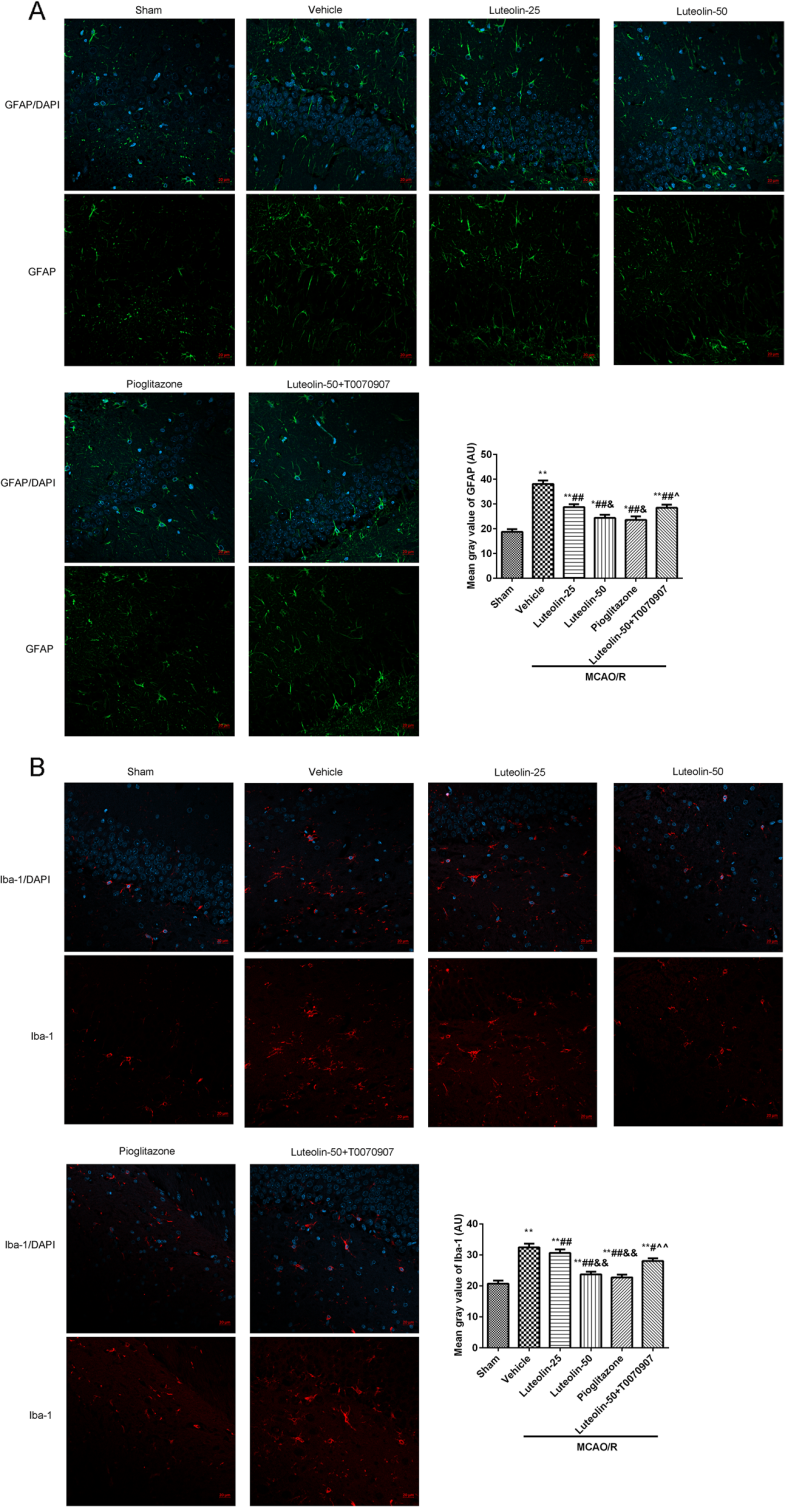
Luteolin reduced hippocampal neuroinflammation through PPARγ in MCAO/R treated rats. The GFAP (**A**) and Iba-1 (**B**) expression levels in damaged CA1 hippocampal region were observed using immunofluorescence, Scar bar = 20 μm. The mean gray value counted through Image J software. Vs. sham group, ^**^p < 0.01; Vs. vehicle group, ^##^*p* < 0.01; Vs. luteolin-25 group, ^&^*p* < 0.05, ^&&^*p* < 0.01; Vs. luteolin-50 group, ^^^^*p* < 0.01

The activation of glial cells, astrocytes (GFAP, Fig. 3A and Fig. 4A) and microglial (Iba-1, Fig. 3B and Fig. 4B), were observed in damaged CA1 hippocampal region through immunohistochemistry and immunofluorescence. The GFAP and Iba-1 expression levels were analyzed by Image J software. Compared with the sham group, GFAP and Iba-1 expression in the damaged CA1 hippocampal region were obviously increased after MCAO/R injury. With the increase of luteolin, the expression levels of GFAP and Iba-1 were clearly decreased. At the same time, the PPARγ agonist pioglitazone treatment significantly suppressed the activation of GFAP and Iba-1 caused by MCAO/R injury. However, the PPARγ inhibitor T0070907 hampered the effects of 50 mg/kg luteolin.

### Luteolin reduced vacuolization of mitochondria in damaged hippocampus through PPARγ in MCAO/R treated rats

**Fig. 5:**
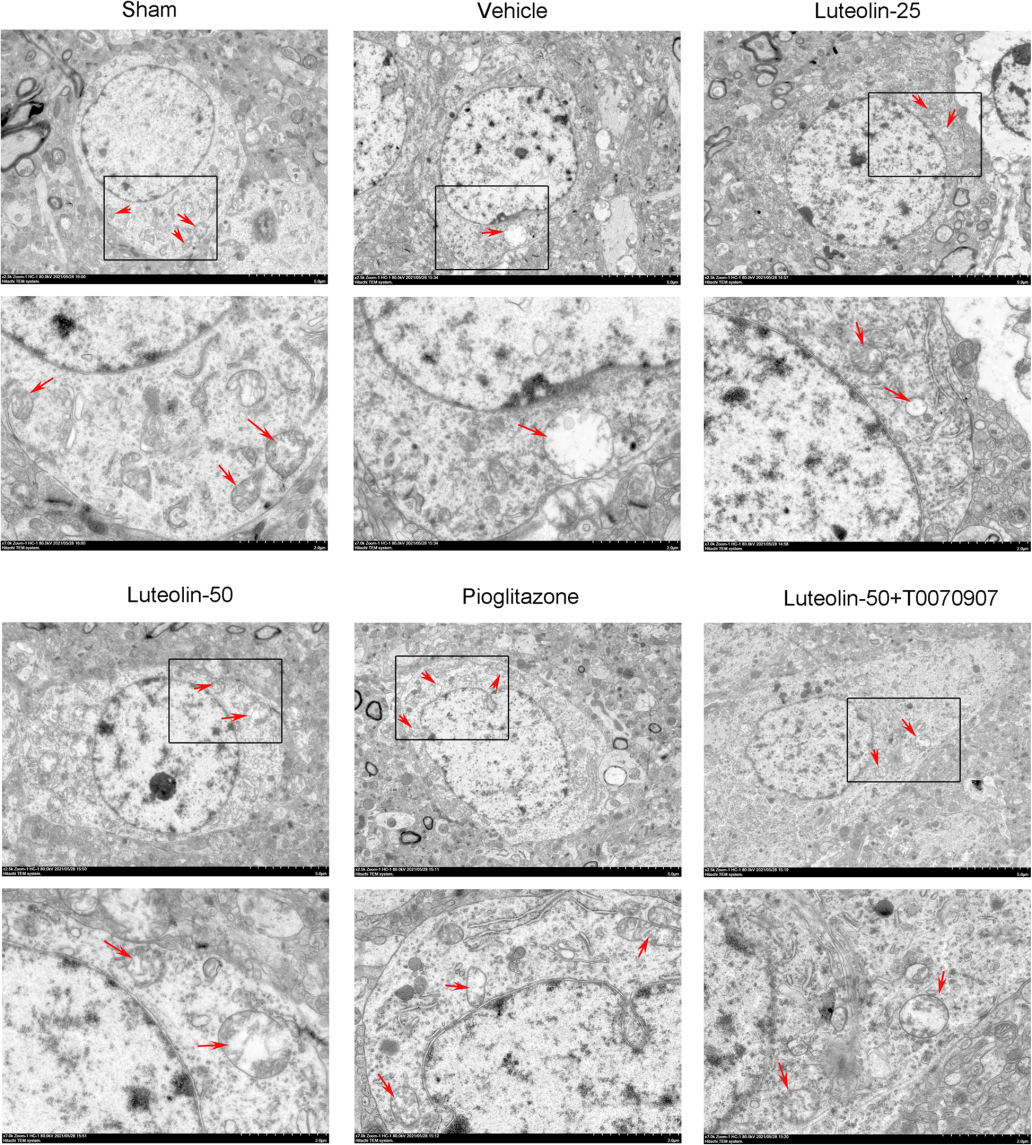
Luteolin reduced vacuolization of mitochondria in damaged hippocampus through PPARγ in MCAO/R treated rats. Red arrows represented mitochondria. Scar bar = 5.0 μm, 2.0 μm

Changes of mitochondria morphology (red arrows) in damaged CA1 hippocampus was observed through TEM (Fig. 5). The structure of mitochondria was obvious and complete in the sham group, while the mitochondria vacuole degenerated in the MCAO/R group. After treatment with luteolin or pioglitazone, the vacuolization of mitochondria was suppressed However, the protective of luteolin (50 mg/kg) were inhibited by T0070907.

### Luteolin reduced LC3B expression in damaged hippocampus through PPARγ in MCAO/R treated rats

**Fig. 6:**
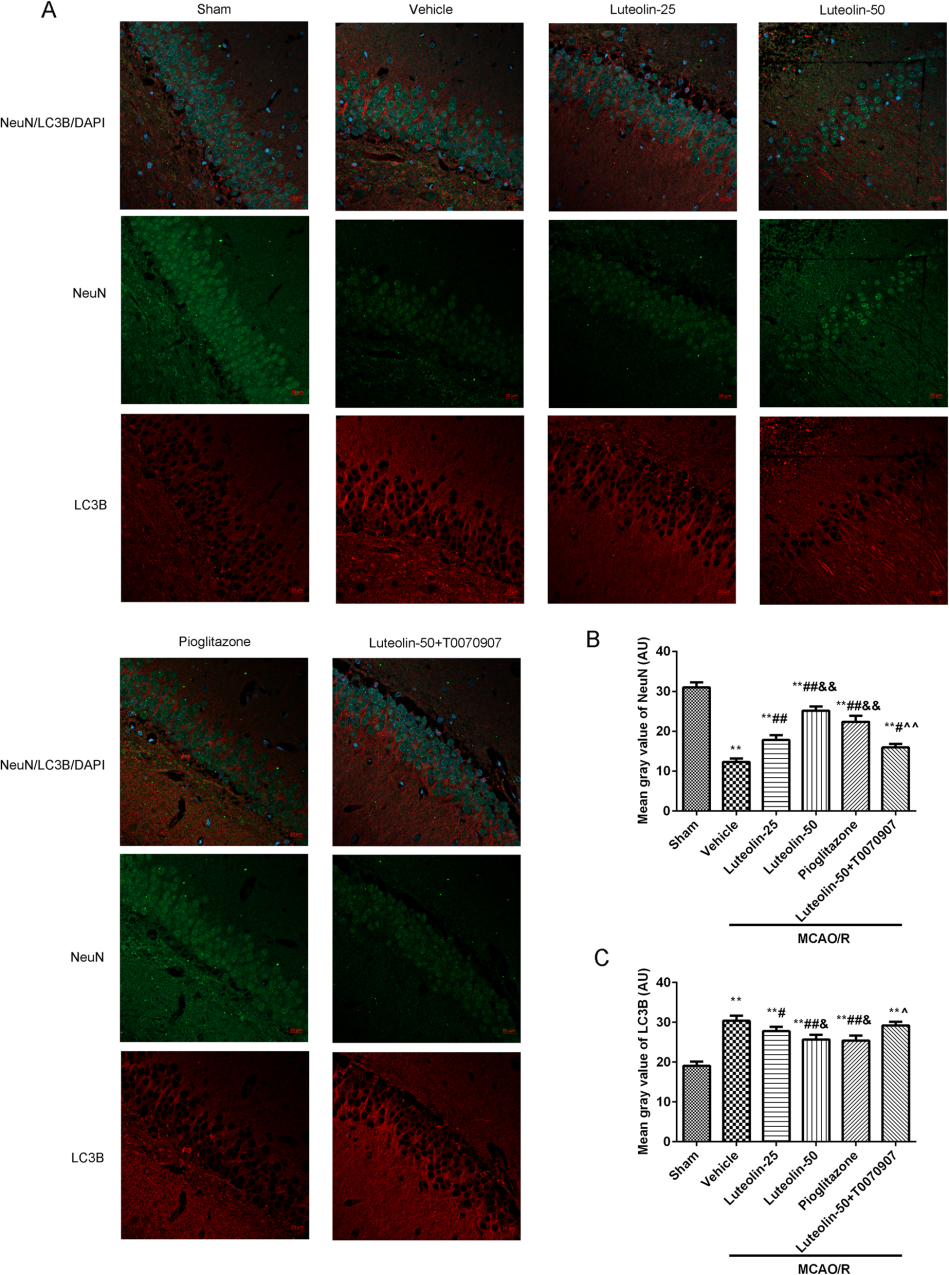
Luteolin reduced LC3B expression in damaged hippocampus through PPARγ in MCAO/R treated rats. **A** The NeuN and LC3B expression levels in damaged CA1 hippocampal region were observed using immunofluorescence, Scar bar = 20 μm. The mean gray values of NeuN (**B**) and LC3B (**C**) were analyzed through Image J software. Vs. sham group, ^**^p < 0.01; Vs. vehicle group, ^#^*p* < 0.05, ^##^*p* < 0.01; Vs. luteolin-25 group, ^&^*p* < 0.05, ^&&^*p* < 0.01; Vs. luteolin-50 group, ^^^*p* < 0.05, ^^^^*p* < 0.01

The expression of NeuN and LC3B in the damaged CA1 hippocampal region was measured through immunofluorescence (Fig. 6). In Fig. 6B, the mean gray value of NeuN was significantly decreased after MCAO/R injury compared with the sham group. After treatment with luteolin or pioglitazone, the mean gray values of NeuN were clearly increased. But, the T0070907 treatment suppressed the roles of luteolin (50 mg/kg). In Fig. 6C, the mean gray of LC3B was notably increased after MCAO/R injury contrasted to the sham group. Luteolin or pioglitazone administration inhibited the mean gray values of LC3B compared with the vehicle group. The PPARγ inhibitor T0070907 masked the protection of luteolin (50 mg/kg) on the MCAO/R injury.

### Luteolin activated p-PPARγ and suppressed LC3B and p-NF-κB p65 proteins in damaged hippocampus in MCAO/R treated rats

**Fig. 7:**
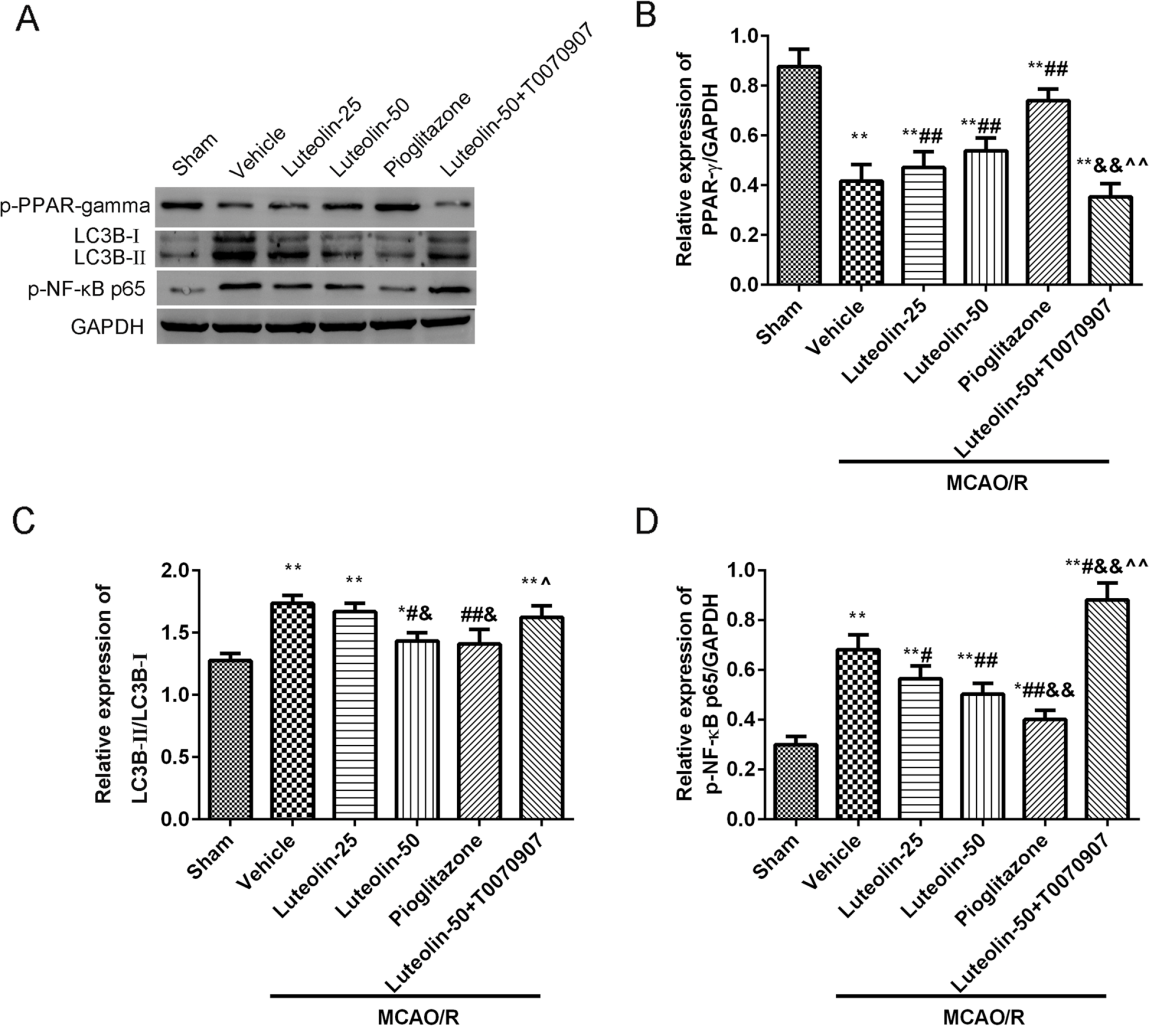
Luteolin increased p-PPARγ and suppressed LC3B and p-NF-κB p65 proteins in the damaged hippocampus in MCAO/R treated rats. **A** The expression levels of p-PPARγ, LC3B and p-NF-κB p65 proteins were tested using western blot. The relative expression of p-PPARγ (**B**), LC3B-II/LC3B-I (**C**), and p-NF-κB p65 (**D**) in the damaged hippocampal region was analyzed via Image J software. Vs. sham group, ^*^p < 0.05, ^**^p < 0.01; Vs. vehicle group, ^#^*p* < 0.05, ^##^*p* < 0.01; Vs. luteolin-25 group, ^&^*p* < 0.05, ^&&^*p* < 0.01; Vs. luteolin-50 group, ^^^*p* < 0.05, ^^^^*p* < 0.01

The proteins expression levels of p-PPARγ, LC3B-II/LC3B-I and p-NF-κB p65 in the damaged CA1 hippocampus were showed in Fig. 7. The results showed that MCAO/R injury downregulated the levels of p-PPARγ, and upregulated the levels of LC3B-II/LC3B-I and p-NF-κB p65. Additionally, luteolin or pioglitazone treatment clearly increased the expression of p-PPARγ, and decreased the expression of LC3B-II/LC3B-I and p-NF-κB p65. Contrasted to the luteolin-50 group, the above proteins expression was greatly reversed in the luteolin-50+ T0070907 group.

## Discussion

In this research, we verified our hypothesis in rats, that luteolin played a neuroprotective effect on rats with MCAO/R by activating PPARγ. After treating with 25 mg/kg/d or 50 mg/kg/d luteolin for 7 days after MCAO/R injury, the infarct area and pro-inflammatory cytokines levels were significantly decreased. Further experiments revealed that luteolin ameliorated the CA1 hippocampus damage by decreasing glial cells activation and reducing autophagy in MCAO/R treated rats. To clarify whether luteolin plays protective roles by mediating PPARγ in MCAO/R treated rats, we selected the PPARγ agonist pioglitazone and PPARγ antagonist T0070907 for study. Finally, we found that the results of pioglitazone were consistent with the 50 mg/kg/d luteolin, but T0070907 destroyed the reparative effects of 50 mg/kg/d luteolin on MCAO/R injury in rats.

MCAO/R injury could cause extensive immunocyte to enter the brain and activate neuroglial cells to excrete proinflammatory cytokines that take part in the formation and development of brain oedema as well as damage the plerosis of neurons [22, 23]. After stroke, neuroinflammation was exacerbated due to activated microglia that promoted peripheral leukocytes infiltration and proinflammatory cytokines release and adjacent blood vessels engulfment [24]. NF-κB could be activated by proinflammatory cytokines, such as IL-1β, IL-6 and TNF-α [25, 26]. Subunits of NF-κB p65 are integral in mediating the MCAO/R induced inflammatory response, which is a main inducer of inflammation and apoptotic cell death [27, 28]. Our findings also confirmed that the NF-κB pathway participates in the neuroinflammation response induced by MCAO/R injury. It has been reported that luteolin could downregulate inflammatory cytokine production after myocardial I/R injury in diabetic rats [29]. For traumatic brain injury (TBI), luteolin decreased the nuclear accumulation of NF-κB p65 and the production of IL-1β and TNF-α after injury [30]. Here, luteolin treatment exerted anti-inflammatory effects by activating PPARγ in rats with MCAO/R. Here, luteolin treatment exerted anti-inflammatory effects by activating PPARγ in rats with MCAO/R.

Normally, cells remove damaged cell organs and poisonous macromolecules through autophagy, which is a highly conserved cellular degradative process. Basal levels of autophagy are important for maintaining the stability of the intracellular environment, which is essential for neurons function and the survival [6, 31, 32]. Salkar et al. [6] found that autophagy protein LC3 and autophagosomes accumulated in hippocampus japonicus within hours after TBI, and remained rose for at least 1 week. Zhang et al. [31] reported that dysfunctional lysosomal storage is associated with the early burst of autophagy in neurons following MCAO. Mitochondria are organelles that act as the oxidative energy centers and are necessary for cell survival, and autophagosomes eventually fuse with lysosomes to form autolysosomes, which target mitochondria for autophagy clearance [33]. Damaged mitochondria are the sources of toxic ROS, and result in the MCAO/R induced brain injury [33]. In this study, our data demonstrated that LC3B accumulated in the damaged hippocampus after MCAO/R injury, and luteolin treatment alleviated brain injury by regulating autophagy. These results were supported by a previous report that luteolin affects the autophagy process in TBI [30]. PPARγ agonist could stimulate mitochondrial activity and inhibition of PPARγ led to mitochondrial fission and hyperpolarization to increase ROS [34, 35]. This study showed luteolin treatment improved mitochondrial vacuolization and LC3B accumulation in damaged hippocampus by activating PPARγ activity in MCAO/R treated rats, demonstrating a possible mechanism of luteolin treatment on MCAO/R injury.

Luteolin possesses multiple biological and pharmacological activities, including antioxidant and anti-inflammatory actions [36, 37]. For MCAO injury, luteolin treatment attenuates neuroinflammation [12, 38] and oxidant [17] through inhibiting matrix metalloproteinase-9 (MMP9) and NF-κB signaling, increasing NF-E2 related factor (Nrf2) and PPARγ. Activation of PPARγ inhibits the stimulation of NF-κB and the secretion of inflammatory cytokines to attenuate neuronal autophagic death [12]. Consistent with these results, our data showed that luteolin administration suppressed neuro-inflammation and autophagy in the hippocampus after MCAO/R through activating PPARγ. A good activity of luteolin to PPARγ (score = − 8.2) was also confirmed by molecular docking analysis. These findings indicate luteolin as a potential therapeutic agent for MCAO/R injury.

However, the improvement of this repair process and neurological results are limited in this study. Therefore, a valid therapy, such as the drug combination, must be developed to increase the improvement after MCAO/R damage. Additionally, it has reported that luteolin suppressed MCAO induced neuroinflammation through regulating PPARγ/Nrf2/NF-κB pathway in rats [12]. Luteolin promoted the nuclear translocation of Nrf2 following intracerebral hemorrhage in rats [39]. But the relationship between the nuclear translocation of Nrf2 and PPARγ in MCAO/R injury is not unclear. Furthermore, the specific details of luteolin regulating autophagy and affecting mitochondrial function in MCAO/R injury remain unclear. More in-depth study is to be continued.

## Conclusions

This present study found that luteolin attenuated neuroinflammation and autophagy in the damaged hippocampus by activating PPARγ in MCAO/R rats, suggesting that luteolin treatment might be as a useful pharmacological strategy for improving cerebral I/R damage.

## Supplementary Information

**Additional file 1.**

## Data Availability

The datasets generated and/or analysed during the current study are available from the corresponding author or from the https://pan.baidu.com/s/1wzHrcH4oeJtWWM2aJpqkDA.
