# Supplementary figures and images for: Luteolin alleviates inflammation and autophagy of hippocampus induced by cerebral ischemia/reperfusion by activating PPAR gamma in rats
Source: BMC Complement Med Ther. 2022 Jul 1;22:176. doi: 10.1186/s12906-022-03652-8 (PMC9248165; doi:10.1186/s12906-022-03652-8)

Figure 6

p-PPAR-gamma

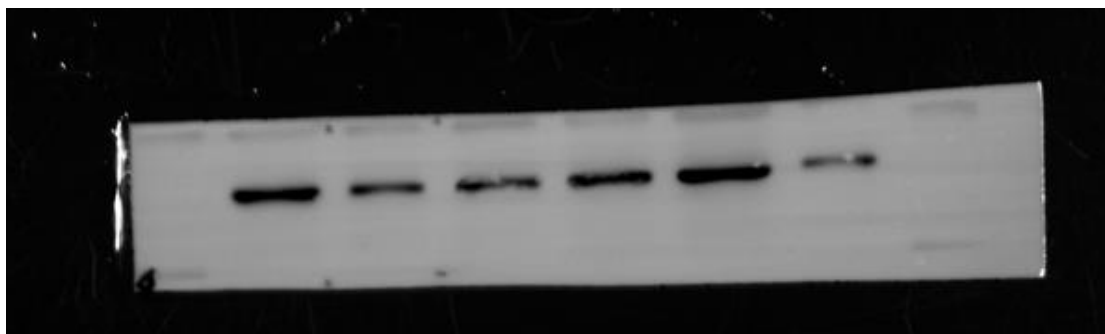

LC3

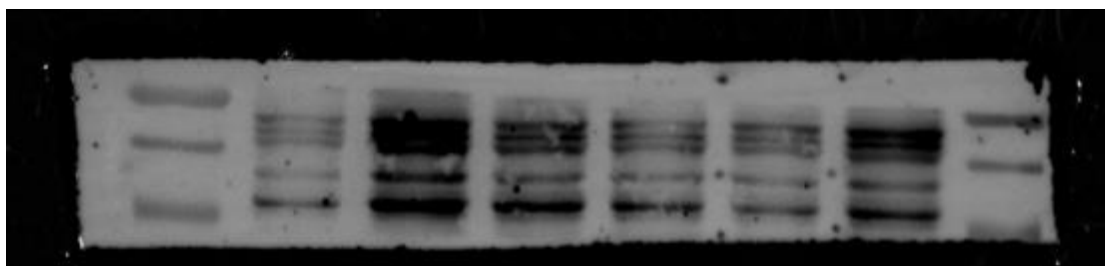

p-NF-kBp65

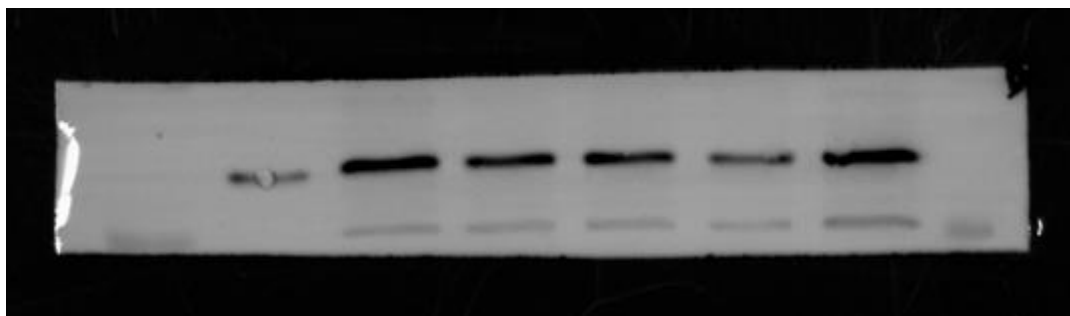

GAPDH

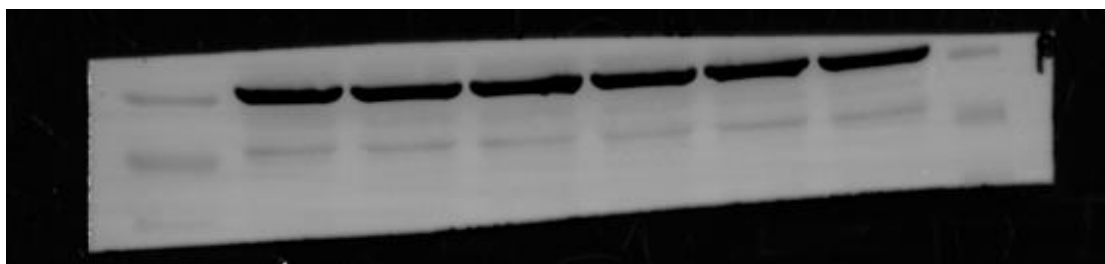

Supplement: Supplementary file 1 — Additional file 1. [file 12906_2022_3652_MOESM1_ESM.pdf]
